# Does Intraoperative Navigation Improve K-Wire Positioning in Reverse Shoulder Arthroplasty?—A New Approach

**DOI:** 10.3390/jpm15110509

**Published:** 2025-10-29

**Authors:** Timo Blaszczyk, Georg Gosheger, Jonathan Wohlmuth, Vincent Hofbauer

**Affiliations:** 1 Department of Orthopedics and Tumor Orthopedics, Muenster University Hospital, Albert-Schweitzer-Campus 1, 48149 Münster, Germany; georg.gosheger@ukmuenster.de (G.G.); vincent.hofbauer@ukmuenster.de (V.H.); 2 Independent Researcher, 52064 Aachen, Germany; jonathan.wohlmuth@rwth-aachen.de

**Keywords:** shoulder, arthroplasty, computer-assisted, navigation-assisted, target system, three-dimensional, CAD, 3D printing, 3D virtual model, personalized medicine, patient-specific

## Abstract

**Background/Objectives:** In reverse shoulder arthroplasty (RSA), precise K-wire positioning of the glenoid component is critical to prevent complications such as glenoid loosening or instability as well as premature implant failure. Optimal component placement must adhere to individualized preoperative plans to account for patient-specific anatomical conditions. Conventional methods often fail to achieve this level of accuracy, undermining the need for personalized medicine. Intraoperative navigation systems are growing in use to improve accuracy in orthopedic surgery. This study aimed to compare the accuracy of K-wire positioning in a 3D-printed model of the scapula using conventional versus navigated methods. **Methods:** We recruited 20 participants: 10 experienced surgeons and 10 inexperienced medical students. Each participant performed four K-wire drillings—two with conventional instruments and two with an intraoperative navigation system. A novel target system, BoneTrack3D, was used to measure accuracy. We assessed the absolute deviation of the entry and exit points as well as the three-dimensional drilling angle. **Results:** The navigated method was significantly more accurate for all measured parameters at a family-wise significance level of α = 0.05. The median absolute deviation for the entry point was 1.6 mm with navigation versus 3.0 mm with the conventional method (*p* < 0.001). Similarly, the exit point deviation was 1.8 mm with navigation versus 6.7 mm conventionally (*p* < 0.001). The drilling angle deviation also showed significant improvement with navigation, at 2.6° compared to 8.9° conventionally (*p* < 0.001). However, the navigated method took longer, with a median drilling time of 100.0 s compared to 55.0 s for the conventional method (*p* < 0.001). The navigated method provided consistent and superior results regardless of a participant’s surgical experience. **Conclusions:** Navigated techniques for K-wire positioning in RSA demonstrate enhanced accuracy in a 3D-printed model, effectively executing a precise, patient-specific preoperative plan. This could be a direct contribution to personalized medicine, ensuring the final implant alignment is tailored to the individual’s anatomy. Furthermore, intraoperative navigation may contribute to a flatter learning curve, thereby increasing accessibility for surgeons with varying levels of experience. Although navigation introduces additional costs and longer initial procedure times, these drawbacks could be offset by improved technical outcomes and a reduced risk of complications. Future studies, including randomized clinical trials and cost-effectiveness analyses, should seek to validate these results in clinical settings with longer follow-up periods and larger patient cohorts to define long-term value and utility of navigation systems in reverse shoulder arthroplasty.

## 1. Introduction

Omarthrosis, or shoulder osteoarthritis, is a degenerative condition that leads to pain, stiffness, and reduced mobility. When conservative treatments fail, surgical joint replacement may be indicated. Options include hemiarthroplasty (replacing only the humeral head), total shoulder arthroplasty (replacing both joint surfaces), and reverse shoulder arthroplasty. Reverse shoulder arthroplasty is a specialized form of shoulder replacement surgery in which the normal anatomy of the shoulder joint is reversed: the ball and socket components of the joint are switched. This means that the ball component is placed on the shoulder blade (glenoid), and the socket component is placed on the upper arm bone (humerus). Through altered biomechanics, this configuration allows the deltoid muscle to compensate for a deficient or torn rotator cuff, restoring some movement and stability to the shoulder. Over the years, reverse shoulder arthroplasty (RSA) has become increasingly important in replacement of the native shoulder joint, especially in cases of rotator cuff deficiency and complex fractures [1,2]. Optimal positioning of prosthetic implants is not a standardized, one-size-fits-all procedure; rather, it must be meticulously tailored to the individual patient’s anatomy, which frequently presents significant challenges. Suboptimal component placement can lead to a range of complications, including instability, scapular notching, impingement, limited range of motion and premature implant failure [3]. Therefore, highly precise, patient-specific placement of the prosthetic implants, which mainly depends on K-wire positioning in the glenoid component, is required. This necessity to accurately translate an individualized preoperative plan into the operation room is the fundamental application of personalized medicine in RSA.

At the same time, intraoperative navigation systems have improved accuracy in orthopedic surgery and are used more frequently in various subspecialties [4,5,6]. Recent studies highlight the impact of computer-assisted navigation in total knee arthroplasty, contributing to more precise implant positioning and alignment [7,8]. In spine surgery, image-guided navigation enables safer and more accurate placement of pedicle screws, reducing complication rates and radiation exposure for both patients and surgical teams [9]. The use of navigation in orthopedic oncology has also grown, allowing for meticulous tumor resections with improved margin control and therefore reduced local recurrence [10]. Furthermore, studies in trauma surgery demonstrate that navigation supports minimally invasive fixation of complex fractures, particularly in anatomically challenging regions such as the pelvis and acetabulum [11,12]. Several studies indicate that navigated RSA allows for more accurate screw placement in the glenoid component compared to conventional RSA, which may help preserve bone stock and reduce the occurrence of complications such as scapular notching [13,14,15]. These findings underscore the growing role of navigation systems as essential tools for achieving higher precision, enhancing patient safety, and facilitating learning curves for both experienced and novice surgeons. However, currently no publications address the accuracy of glenoid K-wire positioning in RSA when comparing conventional and navigated techniques.

The objective of this study was to assess and compare the accuracy of these two methods for glenoid K-wire placement in simulated RSA using a 3D-printed model. Additionally, the operator’s experience and drilling time were analyzed. A 3D-printed model was chosen rather than conventional options such as cadaveric models for several reasons. Cadaveric models can be costly and challenging to obtain, whereas 3D printing offers an alternative that may reduce costs and resource consumption. Moreover, an accurate assessment of K-wire placement using cadaveric models necessitates post-procedural computed tomography (CT) imaging, which is complex to organize and could result in additional expenses as well as possible inaccuracies. Therefore, a novel fast and resource-saving evaluation approach has been employed in our 3D-printed model, as described in the subsequent section.

## 2. Materials and Methods

**Figure 1:**
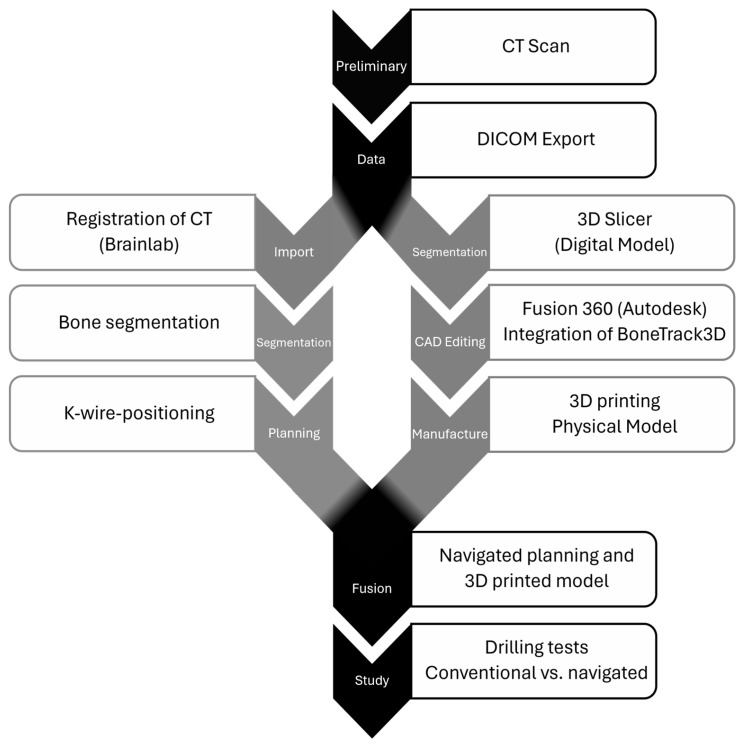
Process flow chart.

To evaluate the accuracy of conventional and navigated K-wire positioning techniques, it was essential to employ an appropriate test model. The comprehensive creation process is depicted in Figure 1 and thoroughly explained in the subsequent sections.

In preparation for subsequent processing, cross-sectional imaging, specifically a CT scan of a shoulder joint in our case, was required. The CT data was then exported in the form of Digital Imaging and Communications in Medicine (DICOM) data and transferred to a segmentation software. Using the free open-source software 3D-Slicer (Version 4.11), the DICOM data was segmented, and the model of the scapula was exported in the format of standard tessellation language (STL) [5,16].

The STL data was first imported into the computer-aided design software (CAD) Fusion 360 (Version V.2.0.13162, Autodesk Inc., San Rafael, CA, USA), which is used to create and process 3D models. Given the scapula’s complexity, the bone surface was initially simplified by reducing the model’s mesh of triangles. The scapula model was then modified to meet the specific requirements. In this instance, a mounting block with screw holes was added to the base of the scapula to facilitate the later setup of the simulated operation. In the following step, BoneTrack3D was integrated. BoneTrack3D is a novel, patent-pending three-dimensional target system which was specifically developed for evaluating precise placement, such as K-wire positioning in our exemplary case. It consists of a frame and two target disks arranged in series, enabling quick and resource-efficient assessment of precise positioning in 3D-printed models. In our case, the target disks were both placed 25 mm apart from each other in series with their center representing the ideal K-wire drill channel in the scapula. The entire experimental design was rooted in the principles of personalized medicine, where the ideal K-wire position was determined based on a CT of a shoulder joint to simulate a precise patient-specific plan. The goal of the drilling tests was not to simply achieve good placement, but to assess the ability of each technique to reproduce the exact, individualized position and angle determined during preoperative planning.

**Figure 2:**
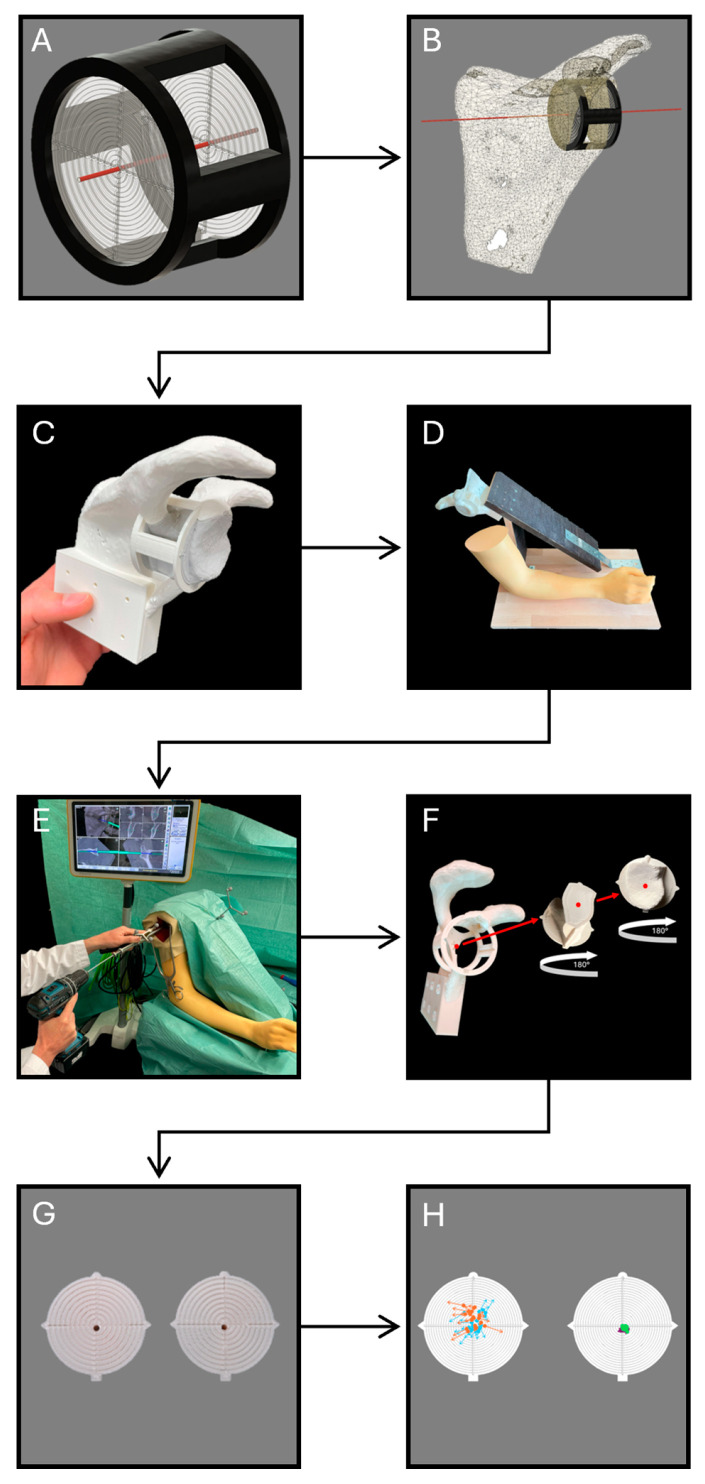
Visualization of the overall process: (**A**) BoneTrack3D; (**B**) scapula model with BoneTrack3D integrated, target disks oriented along the ideal K-wire drill channel (red); (**C**) 3D-printed model; (**D**) raw state of the beach chair simulation model; (**E**) surgeon performing a K-wire drilling using navigated methods; (**F**) exchange of target disks; (**G**) evaluation of the prior K-wire drilling; (**H**) two-dimensional depiction of the three-dimensional drill channel.

The modified scapula model, along with the interchangeable target disks and the intermediate bone stock, were subsequently 3D-printed using an Ultimaker S5 printer (Ultimaker B.V., Utrecht, The Netherlands). The printer is equipped with dual print heads and utilizes polylactic acid (PLA) polymer as well as polyvinyl alcohol (PVA) to simultaneously print the model and its support structures. This feature helps to reduce print time and improves overall print quality. After printing, the target disks were inserted into the scapula model. To simulate beach chair positioning, the modified scapula model was then screwed to an angled wooden plate with the integrated mounting block. The build was securely attached to a table and covered with surgical drapes, mimicking the standard surgical preparation used in the operating room (Figure 2).

Concurrently, the DICOM data was imported into the navigation planning software (Elements version 6.0, Brainlab, Munich, Germany), where the perfect K-wire position was digitally aligned according to preoperative planning.

In the subsequent step, the 3D-printed model was verified against the navigated planning in reference to its accordance with the CT image data. The registration was performed by surface matching, which is based on the manual acquisition of a point cloud on the bone surface.

Once the correspondence between the 3D-printed model and the navigated planning was confirmed, drilling tests could be conducted. Based on the results of preliminary tests and a prior power analysis, 20 participants were recruited for this study: 10 experienced surgeons with at least three years of surgical practice, and 10 inexperienced medical students with no prior surgical experience. Medical students were chosen rather than, for instance, orthopedic residents, as they have sufficient medical knowledge to comprehend the experimental design yet do not possess any surgical experience. Consequently, this categorizes them as absolute novices in this context, which facilitates a distinct separation between subgroups and thereby clarifies the impact of navigation. All participants were provided with an introduction to the experimental setup and given a detailed explanation of optimal K-wire placement based on preoperative planning. Time was allocated for questions. Each participant subsequently performed four consecutive K-wire drillings—completing the initial two procedures using conventional surgical instruments (Medacta, Castel San Pietro, Switzerland), followed by two procedures utilizing intraoperative navigation assistance (Brainlab, Munich, Germany). Additionally, participants did not obtain any feedback about the placement between attempts. This approach was implemented to minimize potential bias resulting from learning effects. The duration of each drilling was measured from the moment participants picked up the operative instruments, intending to begin drilling, until the completion of K-wire placement. The navigation system was already properly calibrated and prepared for use at the start of each drilling. After each drilling, the target disks were blindly exchanged and subsequently evaluated (Figure 2).

**Figure 3:**
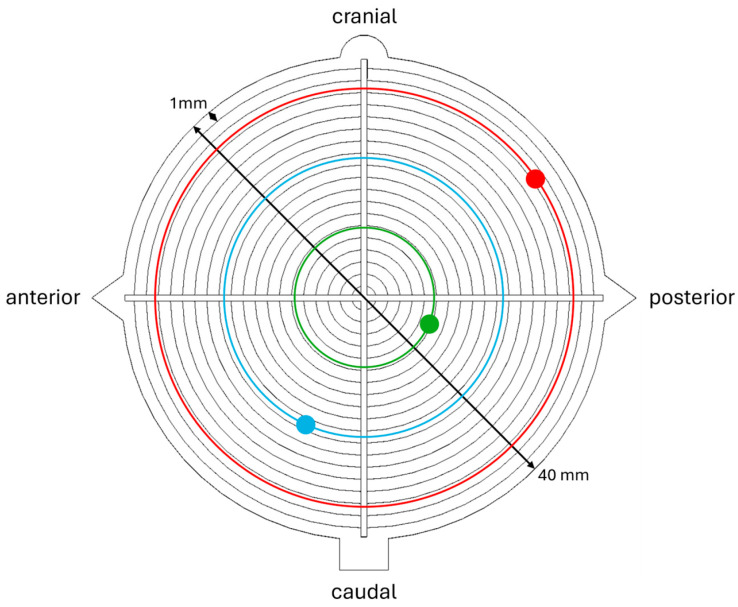
Transfer of the actual drill positions onto concentric rings on the target disk, visualized for three exemplary drill positions (green, blue and red).

For evaluation, each target disk was photographed. The deviation between the ideal drill channel (center of the target) and the actual drill channel on both target disks was measured digitally along the x- and y-axes. To ensure comparability regardless of deviation direction, the absolute values of these deviations were used to map the actual drill positions onto concentric rings centered on the target disks (Figure 3).

**Figure 4:**
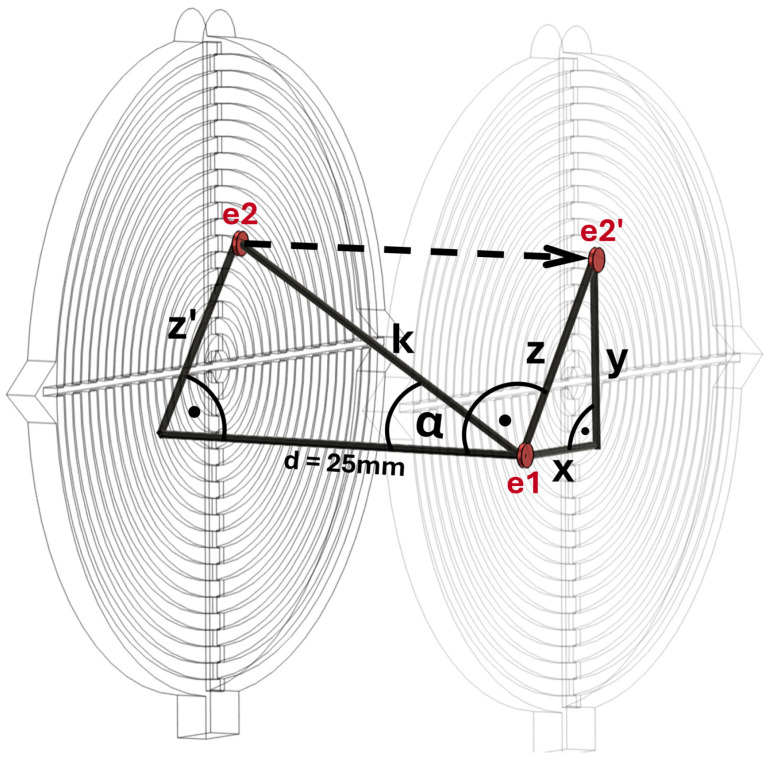
Visualization of the mathematical evaluation: (e1) entry point; (e2) exit point; (e2’) projection of e2 onto the first target disk; (x) distance between e1 and e2’ in the x-axis; (y) distance between e1 and e2’ in the y-axis; (z) direct distance between e1 and e2’; (z’) projection of z onto the second target disk; (d) distance between the target disks = 25 mm; (k) drill channel, direct distance between e1 and e2; (α) three-dimensional drilling angle between e1 and e2.

Figure 4 presents the method used to assess the deviation between the ideal and actual three-dimensional drilling angles. The entry point (e1) and the projection of the exit point onto the first target disk (e2’) are used to calculate the direct distance (z) between e1 and e2’ via the Pythagorean theorem. This value z is subsequently projected onto the second target disk, yielding z’. Utilizing z’ along with the distance between the target disks (d = 25 mm), the parameter k is derived. Finally, the sine function is employed to compute the three-dimensional drilling angle (α) between e1 and e2.

For statistical analysis, the absolute deviation between ideal and actual drill channels on both target disks, the three-dimensional drilling angle and the drilling duration were assessed. The values of both the conventional and navigated drillings of each participant were averaged and used as the basis for further statistical analysis. Entry point and drilling angle were chosen as key parameters to assess K-wire placement accuracy, given their critical influence on the ultimate positioning and alignment of the prosthesis. Precise selection of the entry point ensures optimal placement in reference to the articulating glenoid surface, while accurate drilling angle minimizes deviation from the planned path—both essential for achieving the intended prosthetic fit and biomechanical function [17,18,19,20]. Exit point and procedural time were also measured, serving primarily as supporting metrics in comparing conventional and navigated techniques.

The primary analysis involved the comparison of conventional and navigated techniques, irrespective of participants’ prior surgical experience, using a one-sided paired Wilcoxon signed-rank test. To account for multiple comparisons—specifically four in our case—*p*-values were adjusted using Bonferroni correction, with the family-wise significance level set at α = 0.05. Confidence intervals were calculated at the adjusted level of 98.8% (i.e., 1–α/4), indicating the confidence with which the true median difference between paired observations lies within the given range. The secondary analysis compared results based on participants’ prior surgical experience. As the results for both the inexperienced and experienced groups were not independent, they were analyzed according to the drilling method used. Consequently, no *p*-value adjustment was performed for this comparison. For these analyses, all statistical tests were conducted at a significance level of α = 0.05, and 95% confidence intervals were calculated accordingly.

To reduce potential bias in the study design, several measures were implemented, including structured participant selection and the absence of performance feedback. The order of technique application was standardized: all participants performed conventional K-wire placement before the navigated procedure to limit carry-over effects. K-wire placement accuracy was assessed in a blinded fashion, with evaluators unaware of the technique used or the participant’s identity, thereby minimizing observer bias.

For statistical analysis and interpretation, data were anonymized and analyzed according to predefined objective criteria, including the entry and exit points of the target disks, the three-dimensional drilling angle, and the duration of the drilling procedure. Statistical corrections such as the Bonferroni adjustment for multiple comparisons were applied to control for type I errors. Absolute deviations and confidence intervals were reported for key outcome measures to address the influence of outliers or subjective interpretation. These measures were adopted to minimize bias and support the validity of the study’s conclusions.

## 3. Results

### 3.1. Conventional vs. Navigated (C vs. N)

**Figure 5:**
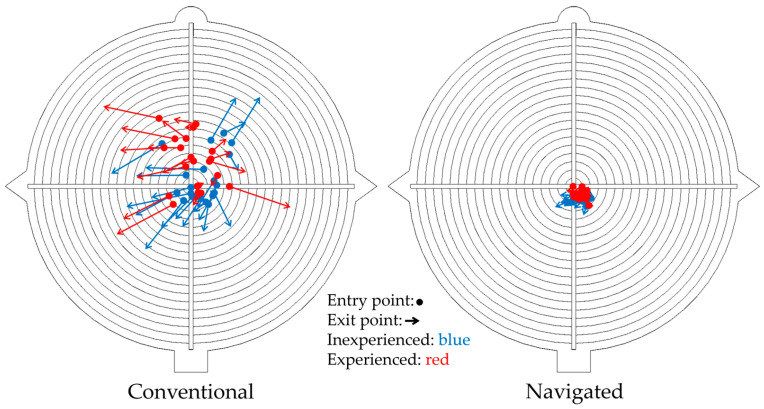
Two-dimensional illustration of the three-dimensional drill channel comparing conventional und navigated techniques. On the left side you can see the drillings performed with conventional methods, on the right side performed with navigated methods. The dots represent the entry point on the first target disk of each individual drilling, while the arrowhead represents the exit point on the second target disk arranged in series. The line in between displays the drilling angle. Drillings performed by inexperienced participants are colored in blue, those performed by experienced participants are colored in red.

The primary research question relates to the comparison of K-wire positioning using conventional (C) and navigated (N) methods regardless of the participants’ prior surgical experience. Table 1 presents the results of this primary comparison. The navigated method demonstrated significantly superior accuracy, with a median absolute deviation of the entry point of 1.6 mm (Q1, Q3: 1.3, 1.8) compared to 3.0 mm (Q1, Q3: 2.2, 4.9) for the conventional method (*p* < 0.001). Similarly, the exit point deviation was substantially lower with the navigated method at 1.8 mm (Q1, Q3: 1.4, 2.0) versus 6.7 mm (Q1, Q3: 5.3, 8.4) conventionally (*p* < 0.001). The drilling angle deviation also showed a significant improvement with navigation, recording 2.6° (Q1, Q3: 1.7, 3.4) compared to 8.9° (Q1, Q3: 7.4, 12.3) for the conventional approach (*p* < 0.001). However, the duration of drilling was longer for the navigated method, with a median of 100.0 s (Q1, Q3: 80.4, 132.0) compared to 55.0 s (Q1, Q3: 37.8, 84.1) for the conventional method (*p* < 0.001). In addition to the statistical analysis, the exact drill channel of each drilling was two-dimensionally illustrated on a target disk (Figure 5).

### 3.2. Inexperienced vs. Experienced (I vs. E)

In the secondary analysis, performance in K-wire positioning depending on the participants’ prior surgical experience was evaluated. The subsequent comparison examines the subgroups as classified by the drilling method employed.

#### 3.2.1. Inexperienced Conventional vs. Experienced Conventional (IC vs. EC)

Table 2 presents the comparison between inexperienced medical students (IC) and experienced surgeons (EC) using conventional methods. For entry point deviation, inexperienced students had a median of 2.5 mm (Q1, Q3: 1.8, 3.8), while experienced surgeons had 4.3 mm (Q1, Q3: 2.9, 5.6), with no statistically significant difference observed (*p* = 0.218). Similarly, exit point deviations were 6.3 mm (Q1, Q3: 5.3, 7.7) for inexperienced students and 6.7 mm (Q1, Q3: 5.9, 9.0) for experienced surgeons, also showing no significant difference (*p* = 0.796). The drilling angle deviation was 10.6° (Q1, Q3: 7.1, 13.5) for inexperienced students and 8.8° (Q1, Q3: 7.9, 10.5) for experienced surgeons, again without a statistically significant difference (*p* = 0.353). Regarding the duration of drilling, inexperienced students took a median of 63.8 s (Q1, Q3: 40.2, 91.0), which was longer than experienced surgeons at 46.2 s (Q1, Q3: 29.8, 77.2) (*p* = 0.571).

#### 3.2.2. Inexperienced Navigated vs. Experienced Navigated (IN vs. EN)

Table 3 presents the comparison between inexperienced medical students (IN) and experienced surgeons (EN) using navigated methods. For entry point deviation, inexperienced students had a median of 1.7 mm (Q1, Q3: 1.6, 1.8), while experienced surgeons had 1.4 mm (Q1, Q3: 1.2, 1.7), with no statistically significant difference observed (*p* = 0.151). However, a statistically significant difference was found in exit point deviations: inexperienced students showed a median of 1.9 mm (Q1, Q3: 1.8, 2.3), which was higher than experienced surgeons at 1.6 mm (Q1, Q3: 1.2, 1.8) (*p* = 0.029). The drilling angle deviation was 2.9° (Q1, Q3: 2.1, 3.5) for inexperienced students and 2.0° (Q1, Q3: 1.3, 3.2) for experienced surgeons, without a statistically significant difference (*p* = 0.315). Regarding the duration of drilling, inexperienced students took a median of 130.0 s (Q1, Q3: 98.8, 146.0), which was longer than experienced surgeons at 86.8 s (Q1, Q3: 54.4, 101.0) (*p* = 0.017).

#### 3.2.3. Inexperienced Navigated vs. Experienced Conventional (IN vs. EC)

Table 4 presents the comparison between inexperienced medical students using navigated methods (IN) and experienced surgeons using conventional methods (EC). For entry point deviation, inexperienced students had a median of 1.7 mm (Q1, Q3: 1.6, 1.8), while experienced surgeons had 4.3 mm (Q1, Q3: 2.9, 5.6), with statistically significant difference observed (*p* = 0.001). Furthermore, a statistically significant difference was found in exit point deviations: inexperienced students showed a median of 1.9 mm (Q1, Q3: 1.8, 2.3), which was lower than experienced surgeons at 6.7 mm (Q1, Q3: 5.9, 9.0) (*p* < 0.001). The drilling angle deviation was 2.9° (Q1, Q3: 2.1, 3.5) for inexperienced students and 8.8° (Q1, Q3: 7.9, 10.5) for experienced surgeons, again with a statistically significant difference (*p* < 0.001). Regarding the duration of drilling, inexperienced students took a median of 130.0 s (Q1, Q3: 98.8, 146.0), which was significantly longer than experienced surgeons at 46.2 s (Q1, Q3: 29.8, 77.2) (*p* = 0.002).

## 4. Discussion

### 4.1. Results of the Study

Our study found that the navigated method demonstrated greater technical accuracy than the conventional method for glenoid K-wire positioning in the simulated operation model. Significantly better accuracy was shown for all characteristics, including entry point, exit point, and the absolute drilling angle when using the navigated method, translating to a potentially more effective implementation of personalized medicine. The superior accuracy may allow the surgeon to respect the complex, often non-neutral, patient-specific version and inclination angles derived from preoperative planning, thereby ensuring the final glenosphere placement is as unique as the patient’s anatomy. Surprisingly, there was no significant difference between the inexperienced and experienced group when using the same method, except for the exit point in navigated methods. A significant distinction was observed, favoring the inexperienced group utilizing navigated methods over the experienced group employing conventional techniques. This indicates that the navigated method may provide consistent excellent results independent of prior experience, suggesting a potentially flatter learning curve and greater accessibility for less seasoned surgeons [21,22]. The ability of navigation to achieve consistent excellent results regardless of experience could also facilitate access to this level of patient-specific care. Especially in the beginning, the duration of drilling is significantly longer with navigation since the surgeons have to get used to a completely new technique. However, this drawback could quickly be compensated by better accuracy and potentially flatter learning curve with progressively shorter drilling times [23].

### 4.2. Strength and Limitations of the Model

In this study, a total of 20 participants were recruited, each performing four K-wire drillings in total. Despite the fact that the statistical difference between the conventional and navigated method was significant, the test group was still pretty small, potentially limiting validity, reliability, and generalizability of our findings.

Although the preoperatively planned drilling involved only 5° of retroversion, 0° of inclination and a 25 mm distance between the target disks, the difference in outcomes between the conventional and navigated groups was substantial. It is anticipated that with more complex planning scenarios and a larger test group, the discrepancy between techniques will become even more pronounced. The beach chair model used in this study was built according to real intraoperative conditions. The printing parameters for the 3D-printed scapula were carefully tailored to closely mimic actual bone tissue, and the density and the internal structure were verified via CT scans. The whole simulation model was endorsed by experienced surgeons as an authentic simulation.

While the beach chair simulation model seems to accurately reflect RSA operation conditions, some differences remain between simulation and actual surgery. The artificial environment remains unable to fully reproduce the complex nuances and unpredictable factors inherent in real surgical procedures. Factors such as the limited bone density, homogeneity of the material, and the absence of patient-specific anatomical variations limit the transferability of the results to real-world clinical situations. Despite the fact that the 3D-printed parts that were drilled into were exchanged after each drilling, repeated use of the model may lead to wear and subtle structural changes, potentially influencing the reproducibility of the drilling experience over time.

Additionally, the placement and stability of the navigation camera as well as the precision of 3D-printed anatomical models play significant roles in the overall accuracy of the system [24]. Issues like warping during printing, segmentation fidelity and decisions regarding reference marker placement can all introduce errors or inconsistencies [25,26]. Although clinical implications cannot be conclusively determined, the standardized environment used in our study does allow for direct comparison of technique-specific outcomes and provides valuable insights into procedural learning curves and technical advantages.

### 4.3. Clinical Relevance

Intraoperative navigation, while a promising tool for enhancing surgical precision, carries inherent limitations and potential complications that require careful consideration. A primary concern is the financial burden, as the high capital cost of the equipment and ongoing maintenance fees can limit its accessibility and economic viability for many healthcare institutions [27]. This is compounded by the prolonged surgical time often required for system setup, patient registration, and recalibration, which can increase the duration of anesthesia and elevate the risk of patient morbidity [28]. The potential for registration errors, where the preoperative imaging data fails to align perfectly with the patient’s anatomy, leading to inaccurate tool tracking, also poses a risk [24]. This can result from patient movement, soft tissue deformation, and inaccuracies in the placement or stability of fiducial markers [29,30]. Finally, while navigation is often employed to facilitate minimally invasive procedures, the necessity of percutaneous pin placement for patient tracking can introduce additional risks, such as infection, iatrogenic fractures or neurovascular injury, which must be weighed against the expected benefits [31,32,33]. These factors highlight the need for a balanced view, acknowledging that the benefits of navigation must be carefully weighed against its inherent costs and risks. These limitations could act as barriers to widespread implementation and adoption.

K-wire positioning in RSA directly impacts the accuracy of glenoid component placement, which is considered extremely important regarding RSA success and functional outcome [34,35,36]. Besides minimizing component malposition, navigation offers further advantages [37]. In the orthopedic patient, compounding factors, such as pre-existing bone defects, severe deformities, and particularly the presence of osteophytes, significantly complicate the orientation for K-wire positioning and therefore glenoid component implantation, even for highly experienced practitioners [38,39]. Bony overgrowths can obscure or alter the reliable anatomical landmarks that surgeons typically use for precise intraoperative assessment of the glenoid vault and the scapular plane. By employing navigated techniques, these patient-specific anatomical challenges are effectively managed, enhancing the precision of component placement in alignment with individualized preoperative planning. With navigation less surgical anatomical exposure is needed, reducing surgical dissection damage. Multiple studies have shown that in navigated RSA fewer and longer screws were inserted than in conventional RSA, therefore saving bone stock [13,14,40,41,42]. This may contribute to improved implant stability and reduced risk of complications as well as better functionality [43,44]. First promising studies show that scapular notching and reoperation is more common in non-navigated than in navigated RSA. Nonetheless, in most studies the follow-up time span is too short for significant differences and further studies with larger cohort sizes are necessary [15].

### 4.4. Further Research

Although our study shows promising results on the superiority of navigation in K-wire placement in a 3D-printed model, further cadaveric and clinical validation is required before definitive conclusions or routine application can be considered. Further studies should seek to validate the clinical relevance of specific thresholds in degrees or millimeters of error and their potential influence on overall outcome. Clinical settings with larger, more diverse patient groups and longer follow-up are needed to evaluate the long-term benefits and complications of navigated K-wire positioning in reverse shoulder arthroplasty. These should include not only technical parameters measuring accuracy but also functional outcomes such as patient-reported outcomes (PROs) and complication rates.

Future research may examine the training effects associated with navigation-assisted techniques in RSA. An experimental design could involve recruiting surgeons to initially perform K-wire drilling on models using conventional methods. Following a structured training period—such as six weeks—utilizing navigation-assisted approaches, the initial test would be repeated to assess and document any improvements in accuracy.

Additional comparative studies examining cost-effectiveness and the impact on surgical training are warranted to guide the integration of navigation technology into routine clinical practice. Furthermore, future investigations could explore advancements in navigation systems, such as the use of augmented reality or artificial intelligence, to further enhance surgical accuracy and efficiency. Addressing these areas will provide a comprehensive understanding of navigation’s role in improving surgical outcomes and inform evidence-based recommendations for its broader adoption.

## 5. Conclusions

In summary, our findings indicate that navigated methods for K-wire positioning in simulated reverse shoulder arthroplasty using a 3D-printed preclinical model result in greater technical accuracy across multiple parameters with less dependency on surgical experience. This may improve component placement accuracy according to patient-specific preoperative planning, therefore directly supporting the application of personalized medicine. Importantly, the controlled environment of our simulation model provided an effective platform for assessing these differences. Despite certain inherent limitations, the model’s realistic composition and immediate feedback on surgical results could benefit surgical training. It offers aspiring surgeons a safe environment with room for error, allowing for ample time for individual development [45,46]. The consistent performance and adaptability of navigated techniques also support their broader implementation in surgical training [47]. Prior to adoption in routine clinical practice, additional cadaveric and clinical investigations are required. Such studies should assess effects on both technical and functional outcomes while also evaluating the potential to reduce complications associated with component malposition. Ongoing research, including randomized clinical trials with longer follow-up periods and larger patient cohorts as well as cost-effectiveness analyses, will be crucial in defining long-term value and utility of navigation systems in reverse shoulder arthroplasty as a core technology for personalized medicine.

## 6. Patents

A patent application (EP 23 17 6689) for the three-dimensional target system BoneTrack3D is currently pending. The inventors of the system are Vincent Hofbauer, Georg Gosheger, and Timo Blaszczyk. Further information is available on the European Patent Office website.

## Figures and Tables

**Table 1:** Comparison of conventional vs. navigated K-wire positioning.

Characteristics	Conventional ^2^	Navigated ^2^	*p* C vs. N [98.8% CI]
Entry point (mm) ^1^	3.0 (2.2, 4.9)	1.6 (1.3, 1.8)	*p* < 0.001 [0.87, ∞]
Exit point (mm) ^1^	6.7 (5.3, 8.4)	1.8 (1.4, 2.0)	*p* < 0.001 [3.7, ∞]
Drilling angle (°)	8.9 (7.4, 12.3)	2.6 (1.7, 3.4)	*p* < 0.001 [4.97, ∞]
Duration of the drilling (s)	55.0 (37.8, 84.1)	100.0 (80.4, 132.0)	*p* < 0.001 [−∞, −21.2]

^1^ The given values represent the absolute deviation of the actual drill positions from the center of the target disks. ^2^ M (Q1, Q3); M = median, Q1 = first quartile, Q3 = third quartile.

**Table 2:** Comparison of conventional K-wire positioning between inexperienced medical students and experienced surgeons.

Characteristics	IC ^2^	EC ^2^	*p* IC vs. EC [95% CI]
Entry point (mm) ^1^	2.5 (1.8, 3.8)	4.3 (2.9, 5.6)	*p* = 0.218 [−3.14, 0.74]
Exit point (mm) ^1^	6.3 (5.3, 7.7)	6.7 (5.9, 9.0)	*p* = 0.796 [−2.98, 2.3]
Drilling angle (°)	10.6 (7.1, 13.5)	8.8 (7.9, 10.5)	*p* = 0.353 [−2.1, 5.69]
Duration of the drilling (s)	63.8 (40.2, 91.0)	46.2 (29.8, 77.2)	*p* = 0.571 [−26.5, 41.0]

^1^ The given values represent the absolute deviation of the actual drill positions from the center of the target disks. ^2^ M (Q1, Q3); M = median, Q1 = first quartile, Q3 = third quartile.

**Table 3:** Comparison of navigated K-wire positioning between inexperienced medical students and experienced surgeons.

Characteristics	IN ^2^	EN ^2^	*p* IN vs. EN [95% CI]
Entry point (mm) ^1^	1.7 (1.6, 1.8)	1.4 (1.2, 1.7)	*p* = 0.151 [−0.12, 0.72]
Exit point (mm) ^1^	1.9 (1.8, 2.3)	1.6 (1.2, 1.8)	*p* = 0.029 [0.06, 1.05]
Drilling angle (°)	2.9 (2.1, 3.5)	2.0 (1.3, 3.2)	*p* = 0.315 [−0.52, 1.73]
Duration of the drilling (s)	130.0 (98.8, 146)	86.8 (54.4, 101.0)	*p* = 0.017 [6.5, 77.5]

^1^ The given values represent the absolute deviation of the actual drill positions from the center of the target disks. ^2^ M (Q1, Q3); M = median, Q1 = first quartile, Q3 = third quartile.

**Table 4:** Comparison of K-wire positioning between inexperienced medical students using navigated and experienced surgeons using conventional methods.

Characteristics	IN ^2^	EC ^2^	*p* IN vs. EC [95% CI]
Entry point (mm) ^1^	1.7 (1.6, 1.8)	4.3 (2.9, 5.6)	*p* = 0.001 [−∞, −1.22]
Exit point (mm) ^1^	1.9 (1.8, 2.3)	6.7 (5.9, 9.0)	*p* < 0.001 [−∞, −3.7]
Drilling angle (°)	2.9 (2.1, 3.5)	8.8 (7.9, 10.5)	*p* < 0.001 [−∞, −4.74]
Duration of the drilling (s)	130.0 (98.8, 146)	46.2 (29.8, 77.2)	*p* = 0.002 [41.5, ∞]

^1^ The given values represent the absolute deviation of the actual drill positions from the center of the target disks. ^2^ M (Q1, Q3); M = median, Q1 = first quartile, Q3 = third quartile.

## Data Availability

The raw data supporting the conclusions of this article will be made available by the authors on request.

## Abbreviations

The following abbreviations are used in this manuscript:

RSA	Reverse shoulder arthroplasty
CT	Computed tomography
DICOM	Digital Imaging and Communications in Medicine
STL	Standard tessellation language
CAD	Computer-aided design
PLA	Polylactic acid
PVA	Polyvinyl alcohol
C	Conventional
N	Navigated
I	Inexperienced
E	Experienced
IC	Inexperienced conventional
EC	Experienced conventional
IN	Inexperiencded navigated
EN	Experienced navigated
CI	Confidence interval
PRO	Patient-reported outcome
